# Establishment of nomogram to predict overall survival and cancer-specific survival of local tumor resection in patients with colorectal cancer liver metastasis with unresectable metastases: a large population-based analysis

**DOI:** 10.1007/s12672-024-01182-y

**Published:** 2024-07-29

**Authors:** Songlin Hou, Lifa Li, Huafang Hou, Tong Zhou, He Zhou

**Affiliations:** 1 The Second Department of Gastrointestinal Surgery, The Affiliated Hospital of North Sichuan Medical College, 1 Maoyuan South Road, Nanchong, 637000 Sichuan People’s Republic of China; 2 Institute of Hepatobiliary, Pancreatic and Intestinal Disease, North Sichuan Medical College, Nanchong, 637000 Sichuan People’s Republic of China

**Keywords:** Nomogram, Colorectal cancer liver metastasis, Local surgery, SEER

## Abstract

**Background and Purpose:**

The tumour-node metastasis (TNM) classification is a common model for evaluating the prognostic value of tumour patients. However, few models have been used to predict the survival outcomes of patients with colorectal cancer liver metastasis (CRLM) with unresectable metastases who received the primary local surgery. Thus, we utilized the Surveillance, Epidemiology, and End Results (SEER) database to establish novel nomograms for predicting the overall survival (OS) and cancer-specific survival (CSS) of these patients.

**Methods:**

Extracted primary data on CRLM patients by local surgery from SEER database. All prognostic factors of OS and CSS were determined by Cox regression analysis. The concordance index (C-index), receiver operating characteristic (ROC) curves and calibration curves were used to further evaluate the accuracy and discrimination of these nomograms. Decision curve analysis (DCA) was executed to evaluate the nomograms for the clinical net benefit. Risk stratification analysis (RSA) was used to evaluate the reliability of them in clinical.

**Results:**

3622 eligible patients were screened and assigned to training cohort (1812) or validation cohort (1810). The age, chemotherapy, tumour grade, primary tumour site, tumour size, lymph node positive rate (LNR), marital status, and carcinoembryonic antigen (CEA) were independent prognostic factors of OS. Additionally, the age, chemotherapy, tumour grade, primary tumour site, tumour size, LNR, and CEA were independent prognostic factors of CSS. The results of C-indexes and ROC curves indicated that the established nomograms exhibited better discrimination power than TNM classification. The calibration curves demonstrated excellent agreement between the predicted and actual survival rates for 1-, 3-, and 5 year OS and CSS. Meanwhile, the validation cohort demonstrated similar results. Background the clinic context, the DCA showed that these nomograms have higher net benefits, and the RSA showed that patients were further divided into low risk, medium risk, and high risk groups according to the predicted scores from nomograms. And, the Kaplan–Meier curve and log-rank test showed that the survival differences among the three groups are statistically significant.

**Conclusions:**

The prognostic nomograms showed very high accuracy, identifiability, and clinical practicality in predicting the OS and CSS of CRLM patients with unresectable metastases treated by local surgery at 1-, 3-, and 5 years, which might improve individualized predictions of survival risks and help clinicians formulate treatment plans.

## Introduction

Colorectal cancer (CRC) is a significant worldwide health problem, and the incidence and mortality are rapidly increasing [1]. The liver is the most common metastatic organ. Approximately 15% ~ 25% of CRC patients have liver metastases at the first time at diagnosis and approximately 25% of CRC patients diagnosed early eventually develop liver metastasis [2, 3]. Moreover, they have only 30 months for the 5 year median overall survival time, and liver metastasis has become the main cause of poor prognosis for CRC patients [4]. Great progress has been made in the systemic treatment of CRLM, but the prognosis of these patients is still not ideal [5, 6]. Surgery remains the preferred treatment strategy for CRLM patients. Synchronous resection for CRLM patients has been gradually applied and popularized due to its advantages of safety and minimal damage, but only 10% ~ 20% of patients are suitable for surgical resection of metastases [7–9]. Thus, for the majority of patients with unresectable metastatic lesions, the advantages of primary lesion surgery are gradually being fully recognized [10, 11]. It has been reported that patients undergoing primary resection can obtain more clinical benefits [12]. Therefore, the treatment and prognosis evaluation of the subgroup of CRLM patients who received primary lesion surgery will be the focus of clinical strategy implementation in the future.

The traditional TNM classification is the most mainstream prognosis evaluation strategy in malignant tumours, and it is stratified according to tumour invasion, lymph node, and organ metastasis [13]. However, this staging system does not offer a reliable prediction of survival for minority-specific patient populations. Up to now, many other prognostic indicators of clinical malignant tumours have been discovered, such as age, sex, tumour size, tumour grade, LNR, and tumour molecular markers, which may contribute considerably to individualized survival prediction. Nomography, as a useful and accessible prognostic tool for physicians, can integrate multiple independent prognostic factors and has been extensively applied to survival prediction, individualized treatment planning, and the follow-up decisions [14–16]. Thus, the established nomogram model has better prediction accuracy than TNM staging systems. However, few studies have employed this model to predict the survival prognosis of CRLM patients who have received local surgery thus far.

The SEER database has a large sample size, high quality, and strong statistical power, which can provide tumor-related researchers with high clinical reference value data [17]. In this study, our purpose was to develop nomograms that estimate the individualized survival probabilities of OS (the overall survival) and CSS (the cancer-specific survival) in CRLM patients treated with primary lesion surgery using data from the SEER database.

## Methods

### Patient selection

Details of CRLM patients were extracted from the SEER database from 2010 to 2015 by utilizing SEER*Stat software (version 8.3.6; http://www.seer.cancer.gov). The SEER database is an authoritative source for surveillance and analysis of various cancers based on the population that collects cancer diagnosis, treatment, and survival data covering approximately 34.6% of the population in the United States [18].

The inclusion criteria were as follows: CRLM patients confirmed by pathology (tissue biopsy) from 2010 to 2015; history of surgical resection for local lesions of CRC; adenocarcinoma histological subtypes according to the standard of the World Health Organization (WHO); primary tumour located in the colorectum; confirmation with liver metastasis; malignant behaviour based on ICD-O-3[(C18,8140/3), (C19,8140/3), (C20,8140/3)]. The exclusion criteria were as follows: primary hepatocellular carcinoma; other metastasis sites outside the liver or combined with other cancers; incomplete clinical, pathological, and follow-up data; and disease confirmed by autopsy or death certificate. All procedures performed in the present study were in accordance with the principles outlined in the 1964 Helsinki Declaration and its later amendments. These data do not require the approval and informed consent of the Institutional review board, because SEER research data is publicly available and all patient data are de-identified.

### Demographics and variables

The demographic variables included sex, age, race, and marital status. The tumour-associated variables included tumour site, tumour stage, tumour grade, tumour size, number of detected lymph nodes, number of positive lymph nodes, number of primary tumours, neurological/venous invasion, CEA, and lymph node ratio (LNR, as the number of positive lymph nodes/number of detected lymph nodes, which is an independent prognostic factor for CRC [19]). The treatment-associated variables included chemotherapy and radiation performance. The survival variables included survival status, cause of death, and survival time. The TNM staging system was applied in line with the 6th edition of the AJCC.

### Nomogram development

The training cohort dataset (n = 1812) from SEER was used to develop the original nomogram. Nomograms were used for visualizing the multivariate regression analysis and predicting 1-, 3-, and 5-year OS and CSS rates. OS was estimated by using the Kaplan–Meier method and compared using the log-rank test, and the difference is shown by the survival curves. The Fine-Gray competing risk model [20] was used to analyse the effect of each variable on the CSS, and the probabilities of cancer-related death and competing risk death were expressed as cumulative incidence functions (CIFs). A multivariate Cox proportional hazard regression model was used in multivariate analysis to identify the independent prognostic factors.

### Validation and calibration of the nomogram

The C-index analysis and the area under the receiver operating characteristic curve (ROC) validated the precision of the prediction model in the training and validation cohorts. The concordance between the predicted and observed probabilities was determined through calibration curve. These prediction models were subjected to bootstrap internal validation. Decision curve analysis (DCA) is a novel algorithm for assessing the clinical utility of various predictive models. It can compensate any limitations of the ROC curve by representing false and true positive fractions as functions of risk thresholds. So, DCA was employed to evaluate the potential clinical application of the novel nomogram. The net benefit of the prediction model in clinical application was evaluated by decision curve analysis. Finally, based on the established models, we conduct prognostic risk stratification analysis (RSA) on patients in the training model group, and further evaluate the prognosis of different risk groups and the clinical reliability of these models.

### Statistical procedures

X-tile software (https://medicine.yale.edu/lab) analysis was applied to determine the optimal grouping cut-off point and complete the transformation of continuous variables to categorical variables. The χ2 test and log-rank test were used to describe the differences between the training cohort and validation cohort in terms of demographic characteristics, tumour characteristics, and treatment characteristics. The hazard ratio (HR) and corresponding 95% confidence interval (95% CI) were calculated. *P-*values less than 0.05 were considered statistically significant. The statistical analyses above were performed by SPSS software (version 22.0) and R software (version 3.6.1, the R packages including rmda, rms, survival, caret, foreign, cmprsk, splines, timeROC and forestplot).

## Results

### Clinical characteristics

**Fig. 1:**
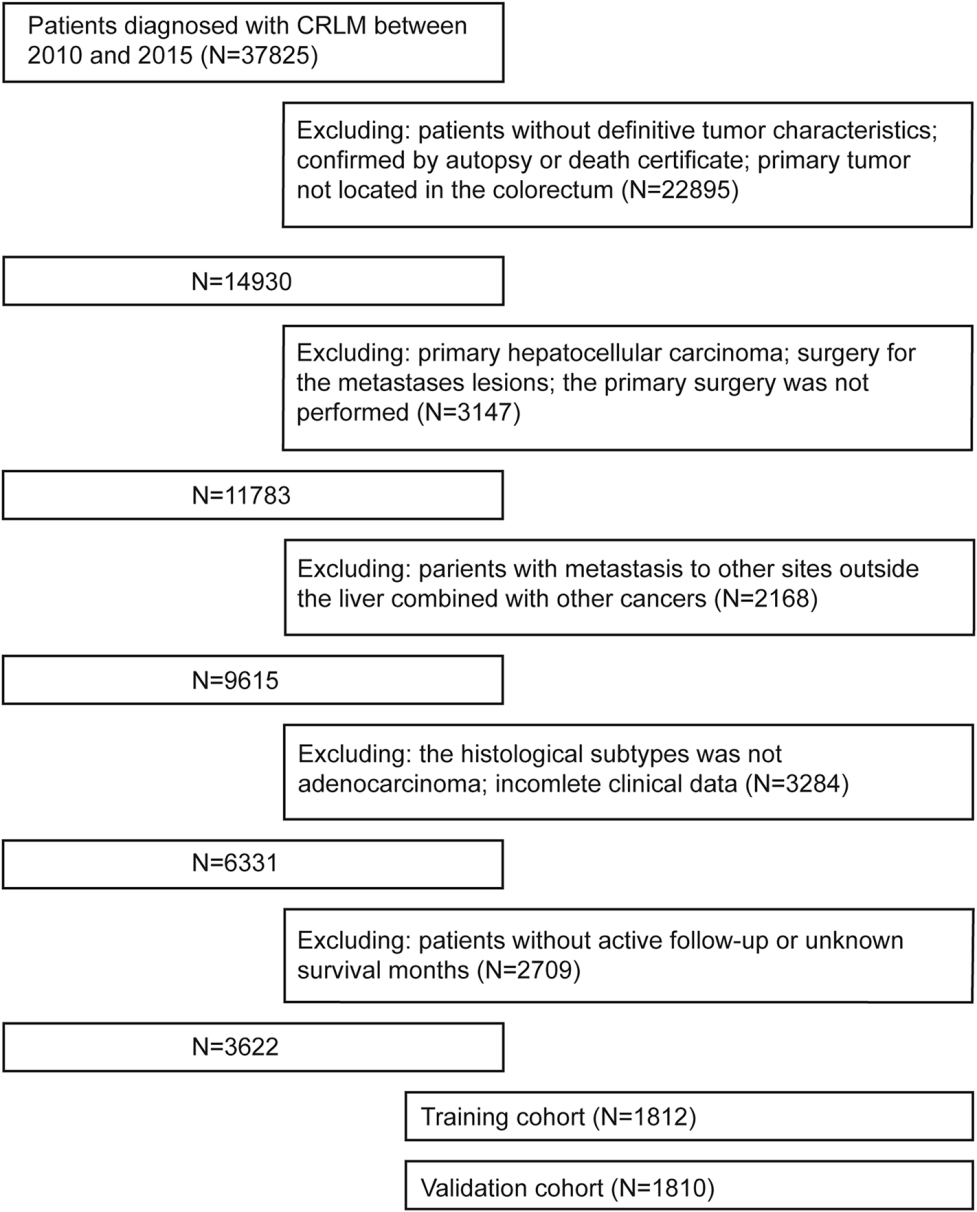
The flow chart of patient selection and grouping process

A total of 3622 CRLM patients who received primary lesion surgery treatment were obtained from the SEER database using the inclusion criteria and were randomly divided into a training cohort (1812 patients) and a validation cohort (1810 patients) at a ratio of 1:1. The patient selection and grouping process is shown in the flow chart (Fig. 1). In the primary cohort, the median age was 61 years old (interquartile range, 52–71 years old), including 1540 females (42.5%) and 2082 males (57.5%), and the median follow-up time was 22 months (interquartile range, 12–36 months). In the training cohort, the median age was 61 years old (interquartile range, 52–71 years old), including 776 females (42.8%) and 1036 males (57.2%), and the median follow-up time was 22.5 months (interquartile range, 12–37 months). In the validation cohort, the median age was 62 years old (interquartile range, 52–71 years old), including 764 females (42.2%) and 1046 males (57.8%), and the median follow-up time was 22 months (interquartile range, 12–35 months). The demographic characteristics and clinical characteristics of the training cohort, validation cohort, and primary cohort are shown in Table 1.

**Table 1:** Demographics characteristics and clinical characteristics of CRLM patients treated by primary lesion surgery in the training cohort, validation cohort, and primary cohort

Variable Factor	Training cohort (N = 1812) No. (%)	Validation cohort (N = 1810) No. (%)	Primary cohort (N = 3622) No. (%)	χ2	*p*-Value
Age(years)				0.792	0.94
18–59	832 (45.9)	805 (44.5)	1637 (45.2)		
60–79	793 (43.8)	810 (44.8)	1603 (44.3)		
80–108	187 (10.3)	195 (10.8)	382 (10.5)		
Sex				0.140	0.93
Female	776 (42.8)	764 (42.2)	1540 (42.5)		
Male	1036 (57.2)	1046 (57.8)	2082 (57.5)		
Race				0.988	0.99
White	1381 (76.3)	1368 (75.6)	2749 (75.9)		
Black	267 (14.7)	281 (15.5)	548 (15.1)		
Asian Pacific	155 (8.6)	150 (8.3)	305 (8.4)		
American Indians	8 (0.4)	11 (0.6)	20 (0.6)		
Grade				0.791	0.99
I (Well differentiated)	71 (3.9)	62 (3.4)	133 (3.7)		
II (Moderately differentiated)	1320 (72.8)	1317 (72.8)	2637 (72.8)		
III (Poorly differentiated)	344 (19.0)	355 (19.6)	699 (19.3)		
IV (Undifferentiated; anaplastic)	77 (4.2)	76 (4.2)	153 (4.2)		
T stage				1.583	0.99
T1	17 (0.9)	16 (0.9)	33 (0.9)		
T2	59 (3.3)	60 (3.3)	119 (3.3)		
T3	1186 (65.5)	1163 (64.3)	2349 (64.9)		
T4	541 (29.9)	557 (30.8)	1098 (30.3)		
Tx	9 (0.5)	14 (0.8)	23 (0.6)		
Tumor site				2.338	0.67
Right hemicolon	846 (46.7)	802 (44.3)	1648 (45.5)		
Left hemicolon	774 (42.7)	817 (45.1)	1591 (43.9)		
Rectum	192 (10.6)	191 (10.6)	383 (10.6)		
Tumor size(mm)				2.643	0.62
2–52	1042 (57.5)	1017 (56.2)	2059 (56.8)		
53–65	356 (19.6)	395 (21.8)	751 (20.7)		
66–920	414 (22.8)	398 (22.0)	812 (22.4)		
Number of primary tumors				1.055	0.90
1	1704 (94.0)	1710 (94.5)	3414 (94.3)		
2	98 (5.4)	87 (4.8)	185 (5.1)		
3	10 (0.6)	13 (0.7)	23 (0.6)		
Number of positive lymph nodes				1.137	0.89
0–1	668 (36.9)	680 (37.6)	1348 (37.2)		
2–7	819 (45.2)	788 (43.5)	1607 (44.4)		
8–44	325 (17.9)	342 (18.9)	667 (18.4)		
LNR				1.680	0.79
< 0.06	578 (31.9)	604 (33.4)	1182 (32.6)		
0.06 ~ 0.52	972 (53.6)	932 (51.5)	1904 (52.6)		
≥ 0.52	262 (14.5)	274 (15.1)	536 (14.8)		
Nerve/vasculature invasion				0.445	0.80
No	1259 (69.5)	1276 (70.5)	2535 (70.0)		
Yes	553 (30.5)	534 (29.5)	1087 (30.0)		
Chemotherapy				1.690	0.43
NP/NA	420 (23.2)	387 (21.4)	807 (22.3)		
Performed	1392 (76.8)	1423 (78.6)	2185 (77.7)		
Radiotherapy				1.453	0.48
NP/NA	1652 (91.2)	1629 (90.0)	3281 (90.6)		
Performed	160 (8.8)	181 (10.0)	341 (9.4)		
Marital				0.489	0.98
Married	1064 (58.7)	1078 (59.6)	2142 (59.1)		
Single/separated	372 (20.5)	355 (19.6)	727 (20.1)		
Divorced/widowed	376 (20.8)	377 (20.8)	753 (20.8)		
CEA				1.999	0.37
Negative	356 (19.6)	390 (21.5)	746 (20.6)		
Positive	1456 (80.4)	1420 (78.5)	2876 (79.4)		

*NP/NA* not performed or unknown

### Prognostic factors associated with OS

**Fig. 2:**
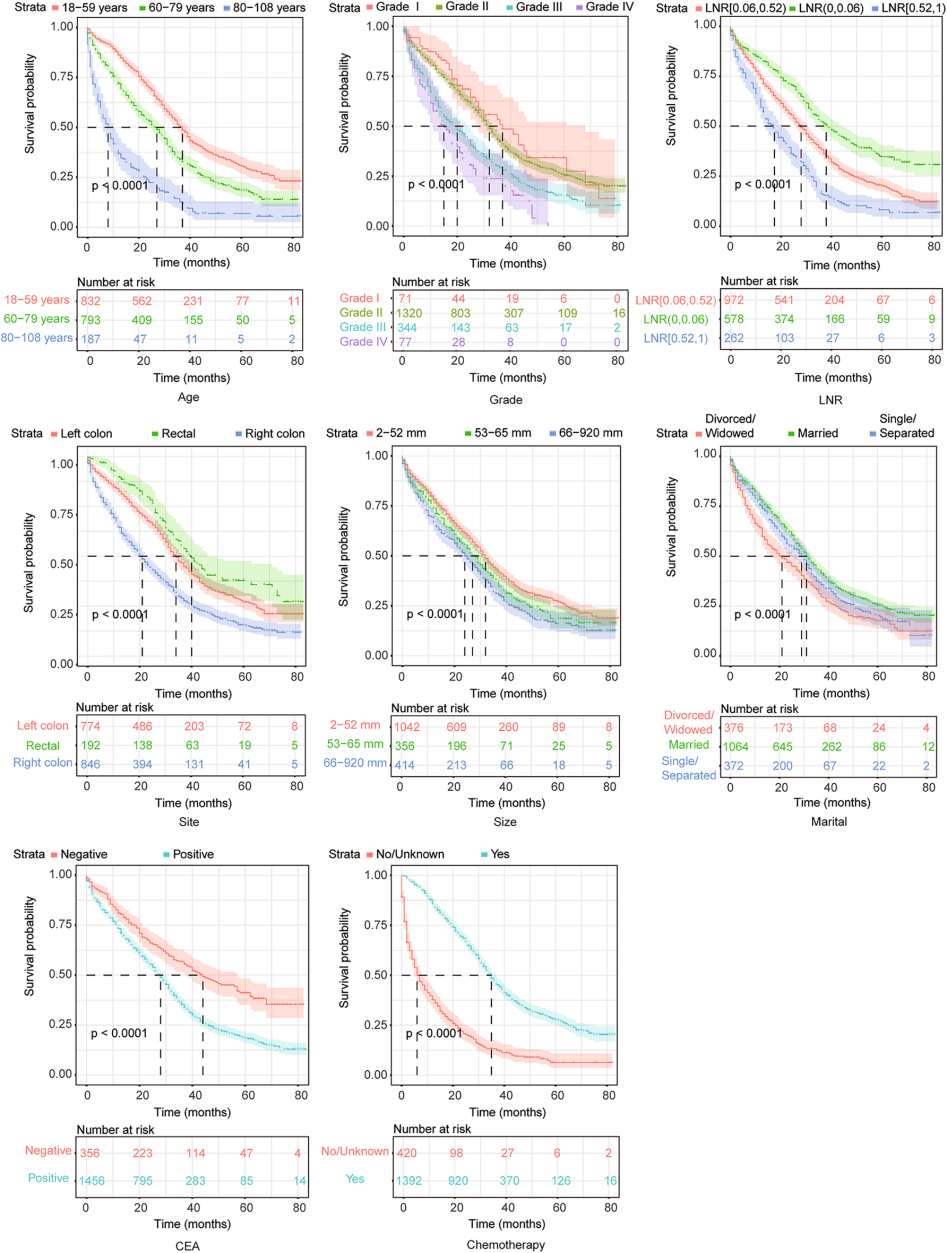
Survival curve analysis of the main factors affecting the postoperative prognosis of patients

In the training cohort, the outcomes of the univariate analysis are shown in Table 2. All significant predictors of OS were assessed by multivariate Cox regression. Combined with the AIC optimization analysis, it was suggested that age, tumour grade, marital status, CEA, LNR, chemotherapy, tumour size, and primary tumour site were independent prognostic factors of OS in patients (Table 2). Based on the Kaplan–Meier and log-rank test methods, the survival curves of each major variable were plotted using the Cox risk model (Fig. 2).

**Table 2:** Univariate and multifactorial survival analysis of postoperative OS for patients

Variable Factor		Single factor		Multi factor	
		HR (95%CI)	*P*-value	HR (95%CI)	*P*-value
Age(years)					
18–59		1		1	
60–79		1.58 (1.39,1.79)	< 0.01	1.34 (1.18,1.53)	< 0.01
80–108		3.73 (3.11,4.47)	< 0.01	2.49 (2.03,3.05)	< 0.01
Sex					
Female		1		–	–
Male		0.95 (0.84,1.06)	0.36	–	–
Race					
White		1			
Black		1.16 (0.99,1.36)	0.06	–	–
Asian Pacific		1.00 (0.81,1.24)	0.99	–	–
American Indians		0.44 (0.14,1.38)	0.16	–	–
Grade					
I (Well differentiated)		1		1	
II (Moderately differentiated)		1.16(0.84,1.61)	0.37	0.96(0.69,1.36)	0.84
III (Poorly differentiated)		1.84(1.31,2.59)	< 0.01	1.44(1.00,2.05)	0.04
IV (Undifferentiated; anaplastic)		2.52(1.68,3.77)	< 0.01	1.99(1.31,3.03)	< 0.01
T stage					
T1		1			
T2		1.51 (0.58,3.93)	0.40	–	–
T3		2.42 (1.01,5.83)	0.04	–	–
T4		3.64 (1.51,8.81)	< 0.01	–	–
Tx		0.83 (0.19,3.48)	0.80		
Tumor site					
Right Hemicolon		1		1	
Left Hemicolon		0.59 (0.53,0.67)	< 0.01	0.70 (0.62,0.80)	< 0.01
Rectum		0.45 (0.36,0.56)	< 0.01	0.62 (0.49,0.78)	< 0.01
Tumor size (mm)					
2–52		1		1	
53–65		1.20 (1.04,1.40)	0.01	1.15 (0.98,1.34)	0.08
66–920		1.40 (1.22,1.61)	< 0.01	1.36 (1.17,1.58)	< 0.01
Number of primary tumors					
1		1			
2		1.035 (0.81,1.33)	0.78	–	–
3		0.87 (0.42,1.84)	0.72	–	–
Number of positive lymph nodes					
0–1		1			
2–7		1.51 (1.32,1.73)	< 0.01	–	–
8–44		2.14 (1.82,2.52)	< 0.01	–	–
LNR					
< 0.06		1		1	
0.06 ~ 0.52		1.60 (1.39,1.84)		1.63 (1.40,1.89)	< 0.01
≥ 0.52		2.66 (2.22,3.18)		2.29 (1.89,2.77)	< 0.01
Nerve/vasculature invasion					
No		1			
Yes		1.18 (1.05,1.34)	< 0.01	–	–
Chemotherapy					
NP/NA		1		1	
Performed		0.27 (0.24,0.31)	< 0.01	0.34(0.29,0.39)	< 0.01
Radiotherapy					
NP/NA		1			
Performed		0.52 (0.41,0.66)	< 0.01	–	–
Marital					
Married		1		1	
Single/separated		1.18 (1.01,1.36)	0.03	1.25 (1.06,1.46)	< 0.01
Divorced/widowed		1.47 (1.28,1.69)	< 0.01	0.92 (0.79,1.08)	0.33
CEA					
Negative		1		1	
Positive		1.76 (1.49,2.07)	< 0.01	1.72 (1.45,2.04)	< 0.01

*NP/NA* not performed or unknown

### Prognostic factors associated with CSS

**Fig. 3:**
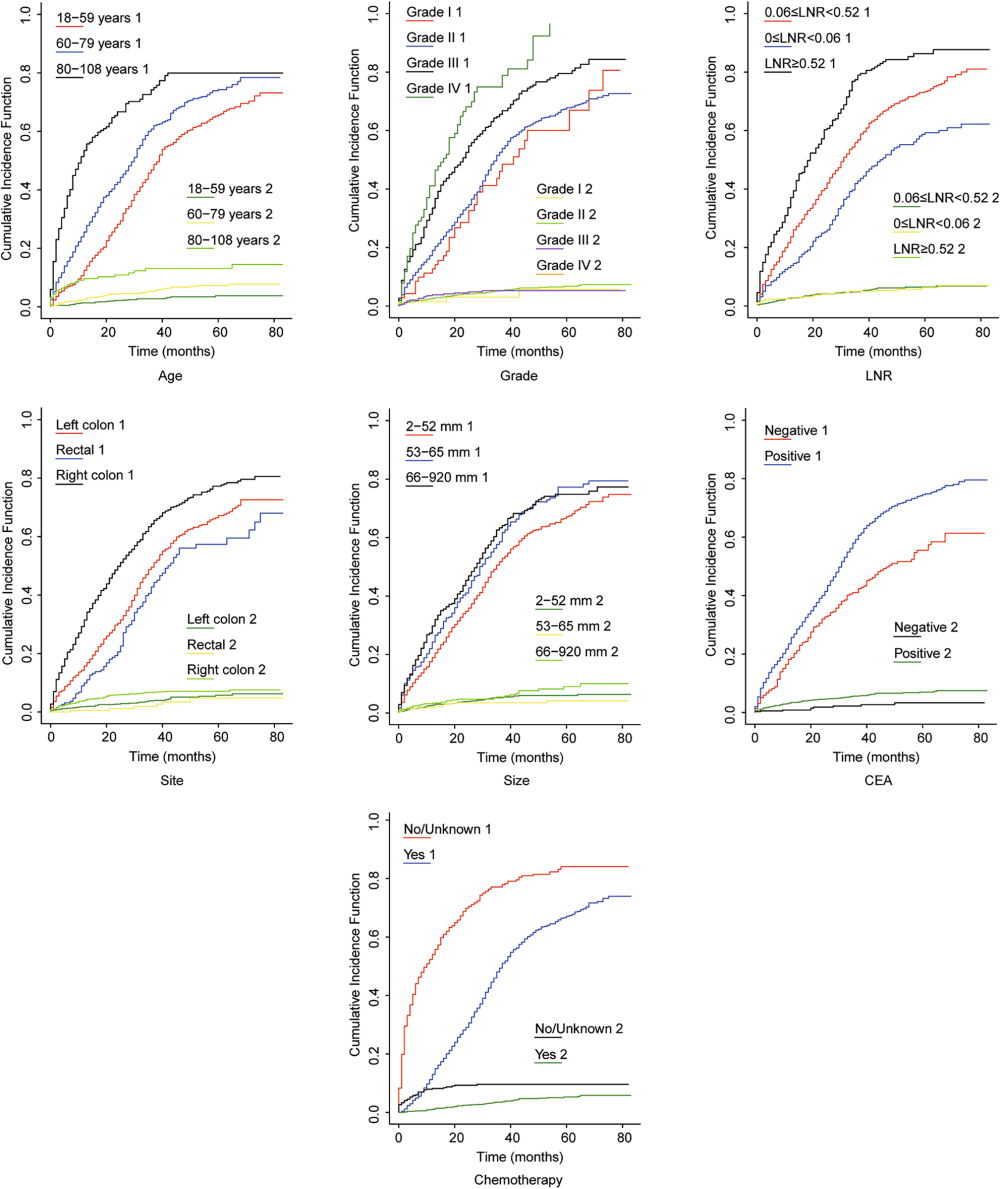
Cumulative incidence curves of postoperative mortality risk in CRLM patients treated by primary lesion surgery in the competing risk model. Number "1" indicates that the cause of death was due to the tumor; number "2" indicates that the cause of death was due to the others

In this study, the Fine-Gray competing risk model was used to conduct independent prognostic factor analyses of CSS, and the multifactorial analysis results with stepwise screening showed that age, tumour grade, CEA, LNR, chemotherapy, tumour size, and tumour site were independent risk factors for CSS in patients (Table 3). The evaluation index of the competing risk model used cumulative incidence function (CIF) analysis (Fig. 3).

**Table 3:** Multifactorial survival analysis of postoperative CSS for patients

Variable factor		Single factor		Multi factor	
		HR (95%CI)	*P*-value	HR (95%CI)	*P*-value
Age(years)					
18–59		1		1	
60–79		1.57 (1.38,1.79)	< 0.01	1.26 (1.10,1.44)	< 0.01
80–108		3.71(3.06,4.51)	< 0.01	1.55 (1.17,2.06)	< 0.01
Sex					
Female		1			
Male		0.95 (0.84,1.08)	0.45	–	–
Race					
White		1			
Black		1.16 (0.99,1.37)	0.07	–	–
Asian Pacific		0.96 (0.76,1.20)	0.70	–	–
American Indians		0.47 (0.15,1.45)	0.18	–	–
Grade					
I (Well differentiated)		1		1	
II (Moderately differentiated)		1.15 (0.8,1.61)	0.42	0.97 (0.73,1.29)	0.83
III (Poorly differentiated)		1.85 (1.30,2.64)	< 0.01	1.33 (0.97,1.83)	0.07
IV (Undifferentiated; anaplastic)		2.67 (1.76,4.05)	< 0.01	1.87 (1.23,2.84)	< 0.01
T stage					
T1		1			
T2		1.63(0.56,4.72)	0.37	–	–
T3		2.76(1.03,7.37)	0.04	–	–
T4		4.30(1.60,11.52)	< 0.01	–	–
Tx		1.00(0.22,4.48)	0.99	–	–
Tumor site					
Right hemicolon		1		1	
Left hemicolon		0.60 (0.53,0.69)	< 0.01	0.77 (0.67,0.88)	< 0.01
Rectum		0.46 (0.37,0.58)	< 0.01	0.70 (0.56,0.87)	< 0.01
Tumor size (mm)					
2–52		1		1	
53–65		1.23 (1.06,1.44)	< 0.01	1.21 (1.02,1.44)	0.03
66–920		1.43 (1.24,1.66)	< 0.01	1.27 (1.08,1.49)	< 0.01
Number of primary tumors					
1		1			
2		0.87 (0.65,1.16)	0.36	–	–
3		0.91 (0.43,1.91)	0.79	–	–
Number of positive lymph nodes					
0–1		1			
2–7		1.56 (1.35,1.80)	< 0.01	–	–
8–44		2.24 (1.89,2.65)	< 0.01	–	–
LNR					
< 0.06		1		1	
0.06 ~ 0.52		1.66 (1.43,1.92)	< 0.01	1.51 (1.29,1.76)	< 0.01
≥ 0.52		2.84 (2.36,3.42)	< 0.01	2.08 (1.68,2.58)	< 0.01
Nerve/vasculature invasion					
No		1			
Yes		1.21 (1.06,1.37)	< 0.01	–	–
Chemotherapy					
NP/NA		1		1	
Performed		0.28 (0.24,0.32)	< 0.01	0.46 (0.38,0.54)	< 0.01
Radiotherapy					
NP/NA		1			
Performed		0.54 (0.42,0.69)	< 0.01	–	–
Marital					
Married		1			
Single/separated		1.19 (1.02,1.38)	0.03	–	–
Divorced/widowed		1.46 (1.26,1.70)	< 0.01	–	–
CEA					
Negative		1		1	
Positive		1.7 3(1.47,2.05)	< 0.01	1.45 (1.22,1.71)	< 0.01

*NP/NA* not performed or unknown

### Construction of the prognostic nomogram

**Fig. 4:**
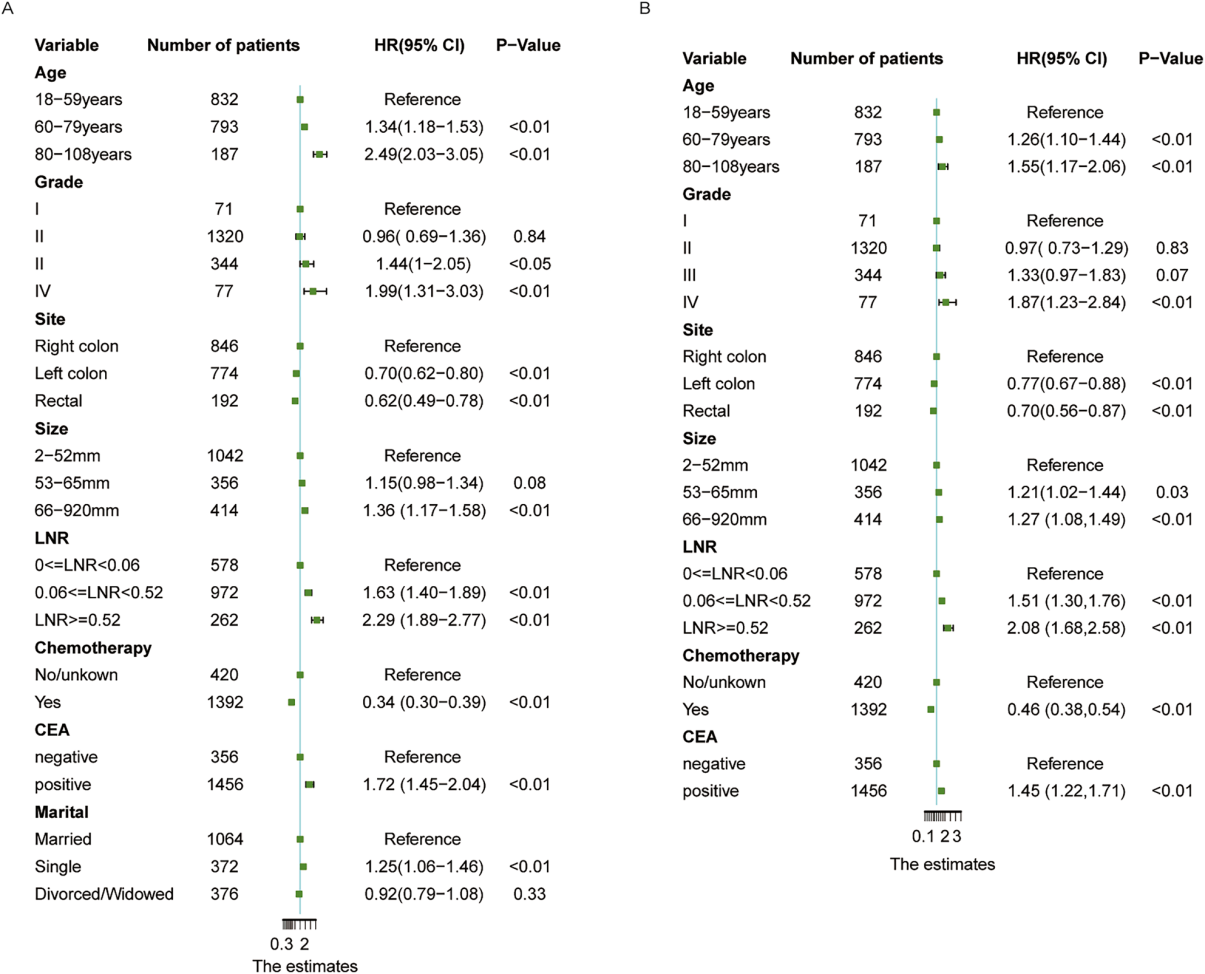
Forest plot of proportional hazards model for OS and CSS in CRLM patients treated by primary lesion surgery. **A** indicates the forest plot of predictors for OS; **B** indicates the forest plot of predictors for CSS

**Fig. 5:**
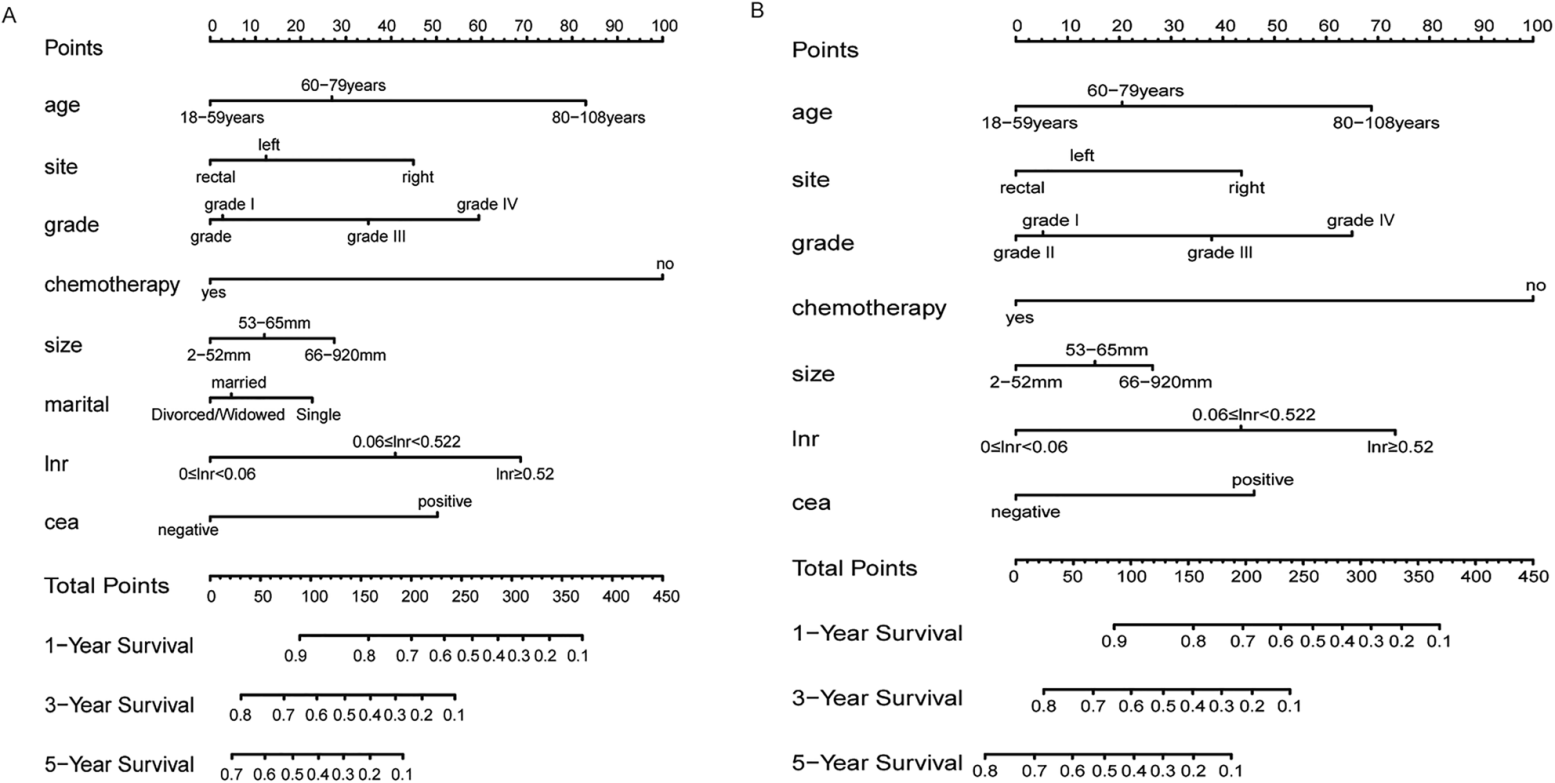
Nomogram prediction models for OS (**A**) and CSS (**B**) at 1, 3 and 5 years in CRLM patients treated by primary lesion surgery

According to the above results, these independent prognostic factors were incorporated into the construction of the nomogram for predicting OS and CSS in the training group (Fig. 4). These nomograms showed the prognostic results at 1, 3, and 5 years for OS and CSS, and the visualization results of the prognostic models are shown in Fig. 5.

### Calibration and validation of the nomogram

In the training cohort and validation cohort, Harrell’s C-index of the prognostic models was [0.747 (95% CI 0.724–0.769) and 0.744 (95% CI 0.721–0.766)] for OS and [0.706 (95% CI 0.682–0.730) and 0.707 (95% CI 0.683–0.0731)] for CSS. These values were considerably greater than those of the TNM staging system, in which the C-indices were [0.614 (95% CI 0.587–0.641) and 0.626 (95% CI 0.599–0.652)] for OS and [0.609 (95% CI 0.583–0.636) and 0.622 (95% CI 0.596–0.648)] for CSS (Table 4). These results ultimately showed the highly discriminative ability to predict the absolute risk for CRLM patients treated by primary lesion surgery.

**Table 4:** C-indexes of the postoperative nomogram models and AJCC-TNM evaluation system for CRLM patients treated by primary lesion surgery

Survival	Evaluation model	Modeling cohort		Validation cohort	
		C-index	95%CI	C-index	95%CI
OS	Nomogram	0.747	0.724–0.769	0.744	0.721–0.766
	AJCC-TNM	0.614	0.587–0.641	0.626	0.599–0.652
CSS	Nomogram	0.706	0.682–0.730	0.707	0.683–0.731
	AJCC-TNM	0.609	0.583–0.636	0.622	0.596–0.648

**Fig. 6:**
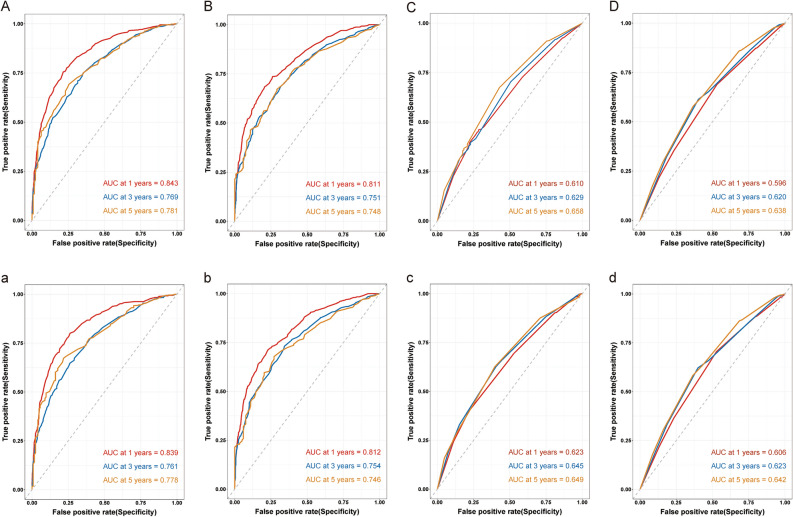
ROC analysis for validating our proposed nomograms for 1 year, 3 years, and 5 years OS and CSS of patients with CRLM treated by primary lesion surgery. **A** and **B** show the AUC for OS in the training and validation cohort in nomogram models; **C** and **D** show the AUC for OS in the training and validation cohort in AJCC-TNM evaluation system; **a** and **b** show the AUC for CSS in the training and validation cohort in nomogram models; **c** and **d** show the AUC for CSS in the training and validation cohort in AJCC-TNM evaluation system

Upon internal validation, we found that the Area under curve (AUC) values of the prognostic nomogram for the 1 year, 3 years and 5 years OS were 0.843, 0.769, and 0.781 in the training cohort, and 0.811, 0.751, and 0.748 in the validation cohort, respectively(Fig. 6A, B); and the AUC values of the prognostic nomogram for the 1-year, 3 years and 5 years CSS were 0.839, 0.761 and 0.778 in the training cohort, and 0.812, 0.754 and 0.746 in the validation cohort, respectively (Fig. 6a, b). Meanwhile, the AUC values for the 1 year, 3 years and 5 years OS predictions in the AJCC-TNM evaluation system were found to be 0.610, 0.623 and 0.658 for the training group and 0.596, 0.620 and 0.638 for the validation group, respectively(Fig. 6C, D); the AUC values for the 1 year, 3 years and 5 years CSS predictions in the AJCC-TNM evaluation system were found to be 0.623, 0.645 and 0.649 for the training cohort and 0.606, 0.623 and 0.642 for the validation cohort, respectively (Fig. 6c, d). It is indicated the superior predictive capabilities of our nomograms.

**Fig. 7:**
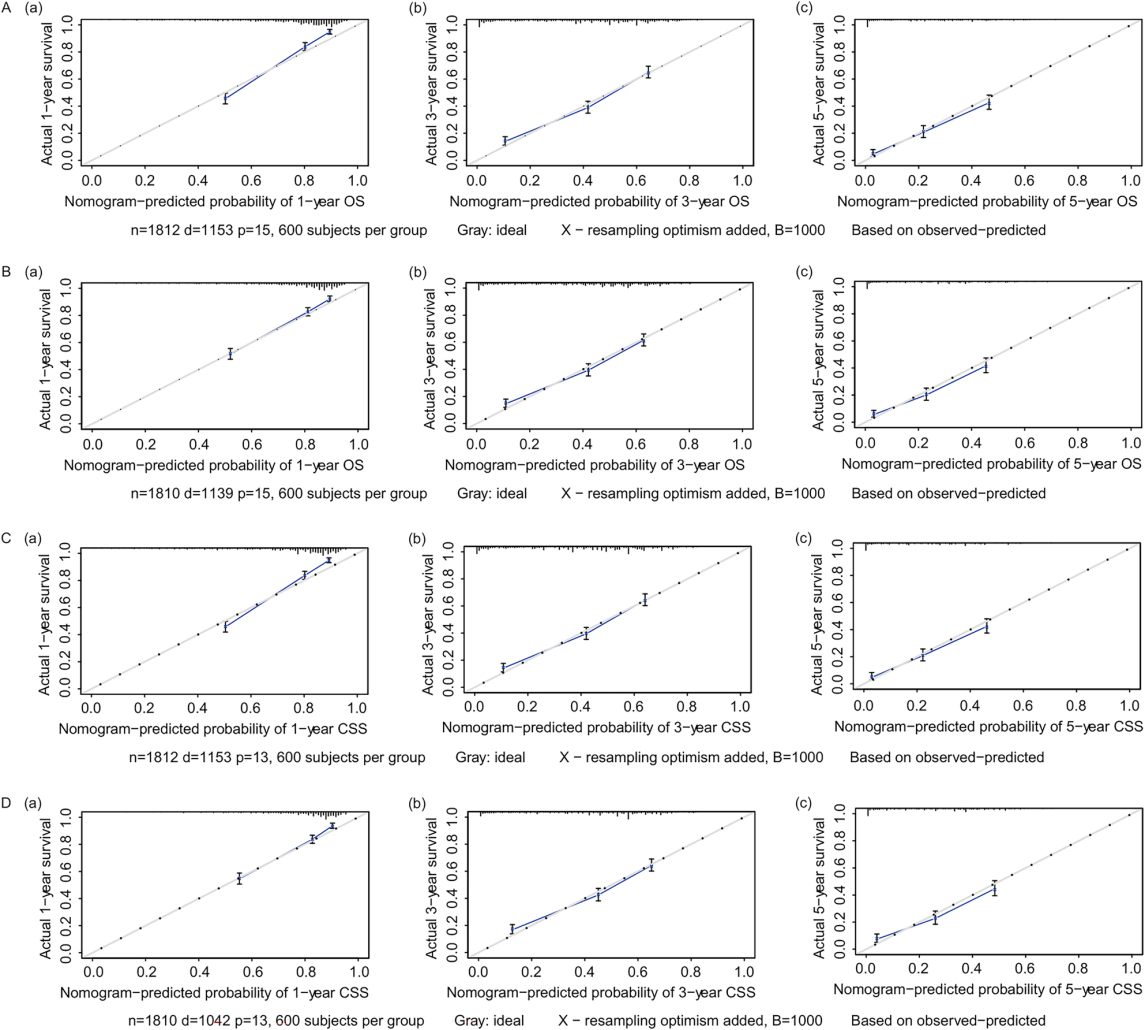
Correction curves of nomogram prediction models for postoperative OS and CSS for CRLM patients treated by primary lesion surgery in the training and validation cohort. **A** (a, b, c) and **B** (a, b, c) show the nomogram model calibration curves for 1 year, 3 year, and 5 year OS for patients in the training and validation cohort, and **C** (a, b, c) and **D** (a, b, c) show the nomogram model calibration curves for 1 year, 3 year, and 5-year CSS for patients in the training and validation cohort. The x-axes showed that the predicted survival was calculated by the nomogram, and the y-axes showed that the actual survival was calculated by the K-M method

To reduce the appearance of overfitting in the C-index, the consistency index was corrected. The calibration curves demonstrated that the predictive power of the nomograms was in line with the actual observed values in both the training and validation cohorts for 1-, 3-, and 5 year OS and CSS (Fig. 7).

**Fig. 8:**
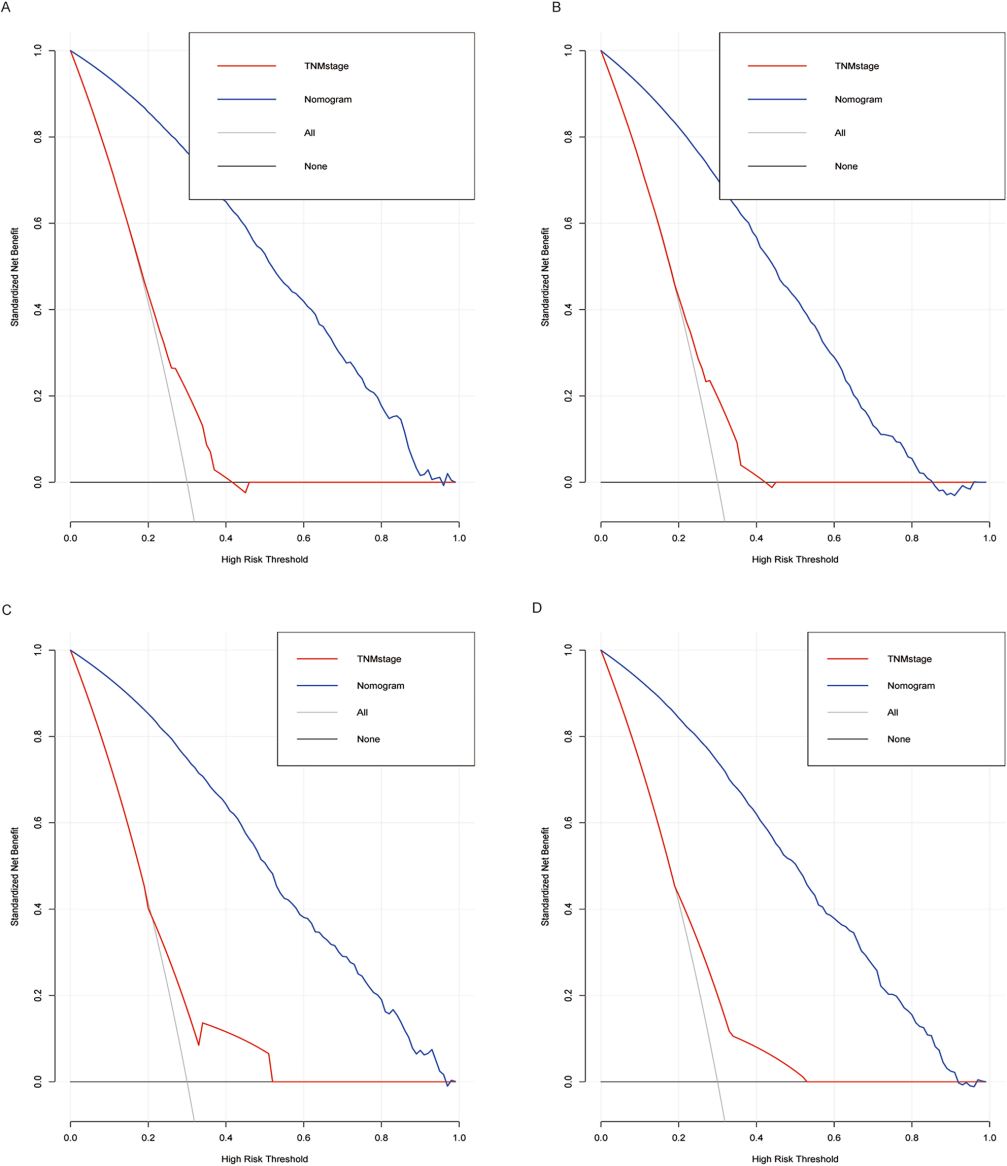
Decision curve analysis for OS (**A,** **C**) and CSS (**B**, **D**) of patients with CRLM treated by primary lesion surgery in (**A**, **B**) training cohort, and in (**C**, **D**) validation cohort.. Black horizontal line indicate that all patients died and did not receive the intervention; gray solid line indicate that no patients died and all patients received the intervention; blue solid line indicate that nomogram model; red solid line indicate that the TNM staging system model

Normally, the DCA curve was widely used to identify the clinical benefts and utility of the nomogram. The clinical net benefits of the nomogram model and the AJCC TNM staging system are presented in Fig. 8. Applying this model to the training cohort and validation cohort, across a broad range of risk thresholds, the blue line (nomogram model) manifests significantly higher net benefits compared to the red line (TNM staging system). For instance, as illustrated in Fig. 8A, at the 30% risk threshold, the net benefit was about 75% in the nomogram model and about 20% in the TNM staging system. That is to say, the nomogram models produced a higher net benefit in assisting the decision to initiate treatment for high-risk patients compared to the TNM staging system or treatment for all patients or none.

### The risk stratification analysis of patient prognosis

In order to further validate the reliability and the accuracy of these models, and to understand the survival risk of the patients in time, so as to make the diagnosis and treatment plans timely, we evaluated the prognosis of the patients through risk stratification analysis. we used the X-tile software, which divided them into low, medium, and high risk subgroups according to their total scores from the nomograms.

Specifically, in terms of OS prognosis, the low-risk group scored 5 ~ 177.5, the median age was 57 years old (interquartile range, 49–65 years old), including 467 females and 677 males, and the median follow-up time was 29 months (interquartile range, 17–43 months); the medium-risk group scored 180 ~ 260, the median age was 65 years old (interquartile range, 58–75 years old), including 172 females and 233 males, and the median follow-up time was 17 months (interquartile range, 9–27 months); and the high-risk group scored 262.5 ~ 437.5, the median age was 75 years old (interquartile range, 64–83 years old), including 137 females and 126 males, and the median follow-up time was 4 months (interquartile range, 1–13 months). Compared with the low risk group, the HR of the medium risk group is 2.62(95%CI: 2.28, 3.01), and the HR of the high risk group is 7.37(95%CI 6.31, 8.62), *P* < 0.01.

**Fig. 9:**
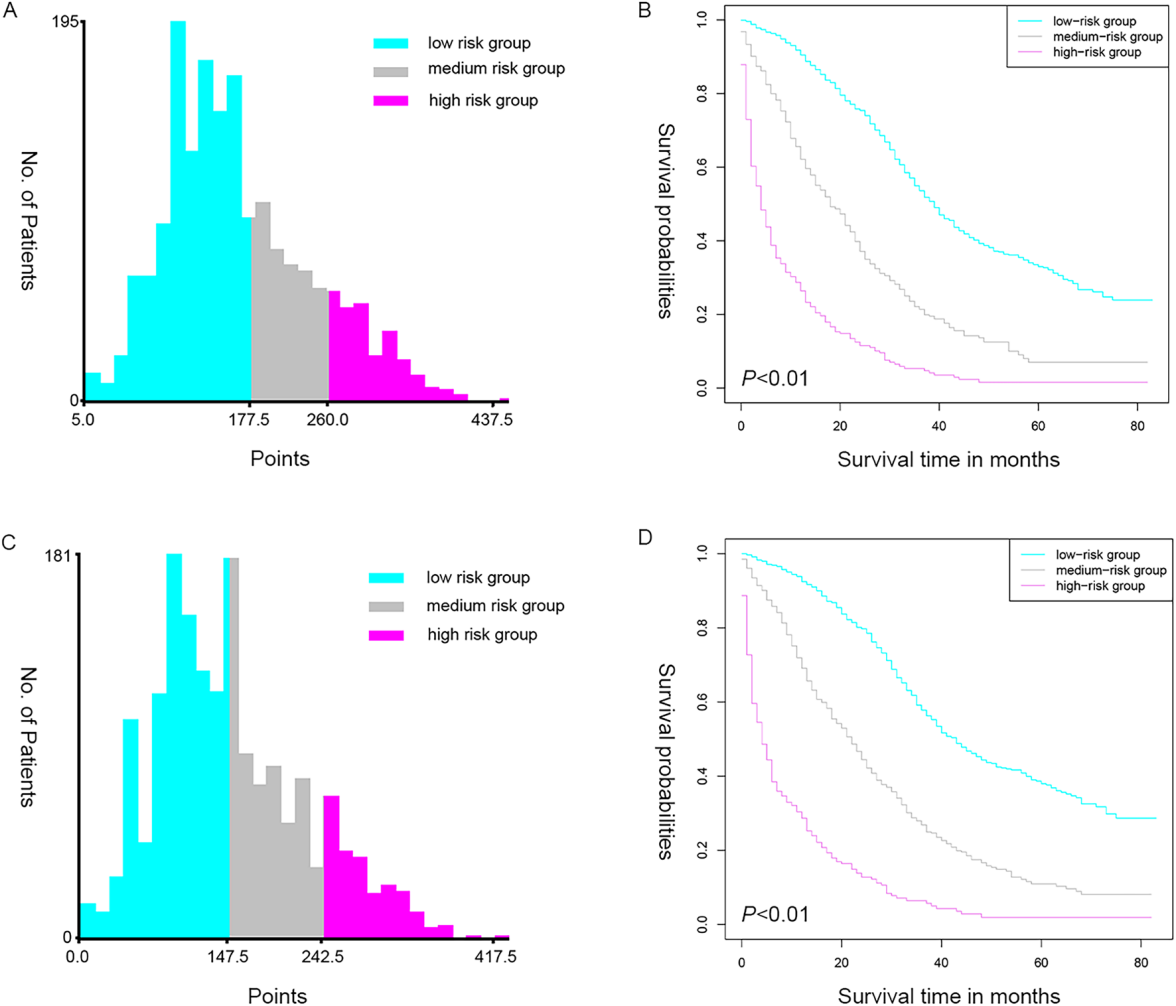
Risk score grouping and survival curve analysis according to these models. **A** and **C** show the risk score group for OS and CSS prognosis, and **B** and **D** show the survival risk of each groups for OS and CSS prognosis

In terms of CSS prognosis, the low-risk group scored 0 ~ 147.5, the median age was 56 years old (interquartile range, 49–63 years old), including 377 females and 561 males, and the median follow-up time was 30 months (interquartile range, 19–44 months); the medium-risk group scored 150 ~ 242.5, the median age was 64 years old (interquartile range, 56–73 years old), including 233 females and 305 males, and the median follow-up time was 18 months (interquartile range, 10–31 months); and the high-risk group scored 245 ~ 417.5, the median age was 75 years old (interquartile range, 63–83 years old), including 124 females and 115 males, and the median follow-up time was 4 months (interquartile range, 1–13 months). Compared with the low risk group, the HR of the medium risk group is 2.57(95%CI 2.42, 2.949), and the HR of the high risk group is 8.21(95%CI 6.93, 9.72), *P* < 0.01. The results showed significant differences among the risk groups, indicating that the established prognostic models have high accuracy and application reliability, and the patients assigned to the low-risk subgroup showed a more favorable prognosis, emphasizing the potential for satisfactory risk stratification of the constructed nomograms (Fig. 9).

## Discussion

Liver metastasis caused by CRC has become a major global health problem and has a poor clinical prognosis [21]. Surgical treatment is still the first choice for CRLM patients, and simultaneous resection is often advocated for to improve the survival and prognosis of these patients [22, 23]. However, for patients with unresectable metastases, a treatment plan is usually negotiated by a multidisciplinary treatment team (MDT). There are many debates surrounding the treatment of primary lesions, but the methods for controlling local tumours have improved survival time in patients with unresectable liver metastases for CRC, including primary lesion resection, radiofrequency ablation (RFA), thermal ablative therapy, and microwave ablation (MWA) [24–26]. Therefore, for CRLM patients undergoing local surgery for primary lesions, it is necessary to acquire accurate prognostic information to help clinicians make better clinical decisions and consultations. Currently, a survival prediction model has not been established for these subgroup patients. Therefore, establishing a practical and convenient survival prediction model for individualized survival evaluation of these patients will be more significant.

A nomogram is a method to present the probability of occurrence of clinical events of statistical prediction models by graphical methods that has the characteristics of high prediction accuracy, flexibility, and simplicity [27]. Nomograms are widely applied to predict the clinical prognosis of cancer patients, such as liver cancer [28], prostate cancer [29], bladder cancer [30], and gastric cancer [31]. In this study, we screened eligible patients from the SEER database and established nomogram models to predict the OS and CSS of CRLM patients by local surgery at 1, 3, and 5 years. These nomograms were composed of a variety of independent prognostic risk factors, including age, grade, size, primary location, chemotherapy, LNR, CEA, and marital status. Compared with the nomogram models of CSS, the marital status risk indicator was included only in the OS prediction model, which highlights the importance of marital status in the evaluation of the overall risk of death of patients.

A prospective cohort study based on randomized clinical trials (RCTs) involving colorectal cancer patients revealed that the disease-free survival (DFS), recurrence-free survival (RFS), and overall survival (OS) of patients who are divorced, separated, or widowed are notably worse than those of married patient [32]. Within the context of cancer, the biological burden of chronic stress may lead to impaired tumour immune surveillance function or DNA damage, and higher adaptive load is associated with an increase in overall cancer-specific mortality rate [33–35]. What’s more, married patients generally exhibit a more positive outlook. The difference in the role of marital status in OS and CSS, it may be that married patients have more life and mental support, which enhances their ability to resist the risk of other diseases. So, when making cancer treatment, the marital status should be considered.

What’s more, in our study, the C-index, ROC curve, calibration curve, and decision curve analysis were applied to evaluate the accuracy of survival prediction and the net benefit in clinical application for the nomograms. First, the calibration curves showed that these nomograms had good accuracy in predicting OS and CSS at 1, 3, or 5 years in both the training and the validation cohorts. Then, the C-index analysis was used to validate the precision of many prediction models, and it assignments greater than 0.7 are widely considered of practical importance. In the study, the C-indices of the nomogram for OS were 0.747 (95% CI 0.724–0.769) in the training cohort and 0.744 (95% CI 0.721–0.766) in the validation cohort. It was superior to the TNM staging system, with C-indices of 0.614 (95% CI 0.587–0.641) in the training cohort and 0.626 (95% CI 0.599–0.652) in the validation cohort. The C-index of the nomogram of CSS was 0.706 (95% CI 0.682–0.730) in the training cohort and 0.707 (95% CI 0.683–0.0731) in the validation cohort. It was superior to the TNM staging system, in which the C-indices were 0.609 (95% CI 0.583–0.636) in the training cohort and 0.622 (95% CI 0.596–0.648) in the validation cohort. Besides, we found that the area under curve (AUC) values of the prognostic nomogram for the 1 year, 3 years and 5 years OS were 0.843, 0.769, and 0.781 in the training cohort, and were 0.811, 0.751, and 0.748 in the validation cohort. The AUC values of the prognostic nomogram for the 1 year, 3 years and 5 years CSS were 0.839, 0.761 and 0.778 in the training cohort, and were 0.812, 0.754 and 0.746 in the validation cohort, respectively. Which all have higher assignments than the AJCC-TNM evaluation system, it is indicated the superior predictive capabilities of our nomograms. In addition, the DCA curves showed that both nomogram prediction models showed a higher net clinic prognostic benefit than the TNM staging system. In order to further clarify the clinical reliability and accuracy of these models, we perform risk scoring on patients in the training cohort, and set high risk, medium risk and low risk groups. The results showed that the risk stratified groups had significant survival differences, indicating that these models have high clinical accuracy and application reliability. It will help to timely identify and judge the survival risk of patients, and make a diagnosis and treatment plan. Above all, these results clearly demonstrated that the established nomograms could successfully predict OS and CSS in CRLM patients with primary lesion surgery at 1, 3 and 5 years and had good discrimination and calibration capabilities. Our study provides the first nomograms that can predict OS and CSS in CRLM patients with unresectable after primary lesion surgery using a large population-based database. These prognostic models provide instruments to inform clinical decisions, such as patient stratifications, consultations, and therapeutic suggestions.

Despite the given advantages of the nomograms presented in this study, there are still some limitations related to this study. First, the nomograms were developed based on retrospective data from the SEER database, and many incomplete cases regarding the population characteristics and pathological information were selectively deleted, which may have resulted in selection bias. Second, the SEER database lacks data about other important prognostic factors, such as the tumour metastasis time, metastasis lesion size, number and therapy of metastatic lesions, specific chemotherapy regimens, comorbidities, and some molecular indicators. In addition, we randomly divided the data selected from the SEER database into the training cohort and validation cohort at a ratio of 1:1. Although this is a common method of nomogram construction and validation, there is no available external verification. Hence, we will still pay attention to the data from multiple medical centres to conduct external cohort validation in the future.

## Conclusions

We developed population-based nomogram prognostic models that can provide individual predictions of OS and CSS for CRLM patients undergoing primary lesion surgery. Providing statistical, usable, and patient-friendly tools will help clinicians more precisely and effectively establish personalized treatment strategies and determine survival prognosis for CRLM patients undergoing primary lesion surgery.

## Data Availability

Data is provided within the manuscript, find some help on our Data availability statements page. The datasets generated and/or analyzed during the current study are available from the SEER database (https://seer.cancer.gov/).
